# Adipocyte and Adipokines Promote a Uterine Leiomyoma Friendly Microenvironment

**DOI:** 10.3390/nu15030715

**Published:** 2023-01-31

**Authors:** Sadia Afrin, Malini Ramaiyer, Umme Aoufa Mafruha Begum, Mostafa A. Borahay

**Affiliations:** 1 Department of Gynecology and Obstetrics, Johns Hopkins University School of Medicine, Baltimore, MD 21205, USA; 2 School of Medicine, Johns Hopkins University, Baltimore, MD 21205, USA; 3 Department of Gynecology and Obstetrics, Khulna City Medical College Hospital, 25-26, KDA Ave., Khulna 9100, Bangladesh

**Keywords:** adipocyte, leptin, leiomyoma, JAK2/STAT3, MAPK/ERK, PI3K/AKT

## Abstract

Uterine leiomyomas are the most common benign tumors of the female reproductive system. Obese individuals have a higher burden of uterine leiomyoma, yet the mechanism relating obesity and leiomyoma development remains unknown. In this study, we observe the effect of adipocyte coculture and leptin treatment on human myometrium and leiomyoma cells. We isolated primary leiomyoma and myometrium cells from hysterectomy or myomectomy patients. Protein expression levels of phosphorylated ERK1/2/total ERK1/2, phosphorylated STAT3/total STAT3, and phosphorylated AKT1/2/3/total AKT1/2/3 were quantified using immunoblotting in immortalized and primary leiomyoma and myometrial cells cocultured with human adipocytes and treated with leptin. An enzyme-linked immunosorbent assay (ELISA) was used to assess pro-inflammatory, fibrotic, and angiogenic factors in immortalized human myometrium and leiomyoma cells treated with leptin. The effects of STAT3, ERK, and AKT inhibitors were assessed in leiomyoma cell lines additionally cultured with adipocytes. Adipocyte coculture and leptin treatment increases the expression of JAK2/STAT3, MAPK/ERK, and PI3K/AKT signaling while inhibitors suppressed this effect. Leptin induces a tumor-friendly microenvironment through upregulation of pro-inflammatory (IFNγ, IL-8, IL-6, GM-CSF, MCP-1, and TNF-α), fibrotic (TGF-β1, TGF-β2, and TGF-β3), and angiogenic (VEGF-A, HGF, and Follistatin) factors in human leiomyoma cells. Furthermore, adipocyte coculture and leptin treatment increases leiomyoma cells growth through activation of MAPK/ERK, JAK2/STAT3, and PI3k/AKT signaling pathways. Finally, STAT3, ERK, and AKT inhibitor treatment suppressed PCNA, TNF-α, TGF-β3, and VEGF-A intracellular staining intensity in both adipocyte coculture and leptin treated leiomyoma cells. These findings suggest that, in obese women, adipocyte secreted hormone or adipocytes may contribute to leiomyoma development and growth by activating leptin receptor signaling pathways.

## 1. Introduction

Uterine leiomyoma, the leading cause for hysterectomies in the United States, are the most common benign neoplasm of the female reproductive system [1]. Uterine leiomyoma present significant economic and medical costs for the United States, with an incidence of over 70% women by the age of 50 and a yearly cost of up to USD 34.4 billion [2]. Early clonal proliferation of uterine myometrium has been identified, in part, as the pathophysiology of uterine leiomyoma, along with deposition of excessive disordered extracellular matrix (ECM). Currently, there are no medical treatment options for individuals with uterine leiomyomas beyond hysterectomy. Therefore, the need to further understand the pathobiology of uterine leiomyoma is underscored by the lack of non-surgical treatment options for individuals with uterine leiomyoma coupled with the large burden of disease.

In recent years, epidemiological studies have determined an association between obesity and the development of uterine leiomyoma, though the underlying mechanism remains unknown [3,4,5,6,7]. Obesity among women aged 18–44 rose to 31.1% in the United States in 2020 [8]. Obesity is defined by large increases in adipose tissue, which has been proven to play endocrine, metabolic, and immune regulatory roles in the body [3]. Adipokines, cytokines released by adipose tissue, have been linked to promotion of tumor growth, through the upregulation of cell proliferation and inflammation [3]. Individuals with leiomyoma have a higher risk of being overweight or obese than without leiomyoma [4]. Recent studies have linked the adipocyte coculture or the secretion of leptin from adipose tissue to the promotion of leiomyoma cell proliferation and ECM deposition [9,10]. Leptin, an adipocyte related hormone, binds to the Ob-Rb receptor and activates MAPK/ERK, JAK2/STAT3, and PI3K/AKT signal transduction pathways. Furthermore, leptin treatment augmented the effect of STAT3 and ERK inhibitors in human leiomyoma cells [9]. These findings suggest that leiomyoma cell proliferation may be leptin-mediated; however, the direct effect of adipose tissue on leiomyoma cells remains unclear. In another study, we observed that adipocyte coculture increased inflammation, fibrosis, and angiogenesis markers in vitro and human tissue, as well as elevated cell proliferation [10].

In this study, we aim to further investigate the pathophysiological association between obesity and uterine leiomyoma development. To do this, we sought to investigate the influence of adipocyte coculture on leptin receptor pathways in human uterine myometrium and leiomyoma cells. We further investigated the potential role of leptin in the inflammation, angiogenesis, and fibrosis in human uterine leiomyoma and myometrium cells.

## 2. Materials and Methods

### 2.1. Cells Isolation and Culture

As previously done, primary myometrium and leiomyoma cells were isolated from five different human patients who underwent hysterectomy or myomectomy at the Department of Gynecology and Obstetrics at Johns Hopkins University Hospital [11,12]. All subjects provided informed consent to participate in the study, which received approval by the Institutional Review Board of Johns Hopkins University. For cell culture, we used Dulbecco’s modified Eagle’s medium/nutrient mixture F-12 (DMEM/F-12) (Thermo Fisher Scientific, Waltham, MA, USA) combined with 10% fetal bovine serum (FBS) and 1% antibiotic–antimycotic in 5% CO_2_ at 37 °C. Human uterine smooth muscle (UtSM) and leiomyoma (HuLM) cells, previously utilized by us and others, were gifted to us by Dr. Darlene Dixon [11,12,13,14]. To culture and maintain the UtSM and HuLM cells, we used a smooth muscle cell growth medium (Lonza, Walkersville, MD, USA), 5% FBS, 0.1% insulin, 0.2% recombinant human fibroblast growth factor B, 0.1% of a mixture of gentamicin sulfate and amphotericin B, and 0.1% human epidermal growth factor in 5% CO_2_ at 37 °C. To model mature adipocytes, we used the human liposarcoma SW872 cell line [15]. We obtained this cell line from American Type Culture Collection (ATCC; Manassas, VA, USA) and used DMEM/F12 with 10% FBS and 1% antibiotic-antimycotic in 5% CO_2_ at 37 °C to maintain the cell line.

### 2.2. Coculture and Treatment

The primary myometrium and leiomyoma cells were treated with 100 ng/mL leptin for 48 h. Human adipocyte (SW872) cells were used to coculture with human primary or immortalized myometrium and leiomyoma cells [10]. We used a Transwell system using 0.4-mm porous membranes from Corning (Corning, New York, NY, USA) to coculture the cells with their respective media. We cultured the Transwell system for eight days, and changed 50% of the medium every 48 h. We then collected the myometrium and leiomyoma media for the multiplex array, and for protein quantification and analysis, we harvested the cells. For a separate series of experiments, the cultured single cells and adipocyte cocultured cells for 7 days were treated with the STAT3 inhibitor (AG490, Calbiochem, Merck Millipore, Burlington, MA, USA) at 50 μM, ERK the inhibitor (PD98059, Cell signaling, Danvers, MA, USA) at 10 μM, and the AKT inhibitor (MK2206, Cayman Chemicals, Ann Arbor, MI, USA) at 10 μM for 24 h. For the leptin treatment, we treated the cells with 100 ng/mL leptin for the first 24 h and then the combination with inhibitors for another 24 h. After 24 h of incubation, we harvested the cells for protein quantification and analysis.

### 2.3. Multiplex Cytokine Array

The medium was gathered from various experiments, centrifuged, and stored at −80 °C. For analysis, we then shipped the media to Eve Technologies Corporation (Calgary, AB, Canada). We used a Multiplex Immunoassay (BioPlex 200; Bio-Rad Laboratories, Hercules, CA, USA) to measure pro-inflammatory, fibrosis, and angiogenesis factors. The sensitivity threshold for an ELISA is 2.2 pg/mL, and coefficient of variation for intra- and inter-assays were 6.3% and 8.1%, respectively.

### 2.4. Western Blot Analyses

Following coculture and leptin treatment, we harvested and lysed the primary and immortalized myometrium and leiomyoma cells in an ice-cold lysis buffer (radioimmunoprecipitation assay buffer; MilliporeSigma, Burlington, MA, USA). The buffer was composed of a phosphatase and protease inhibitor cocktail (MilliporeSigma). We combined the same amounts of protein lysates with 4–12% Bis-Tris gradient gels (Thermo Fisher Scientific) and transferred them onto nitrocellulose membranes (Thermo Fisher Scientific). For 1 h at room temperature, we blocked the membranes by soaking them in Tris-buffered saline with 0.1% Tween-20 (TBST; Thermo Fisher Scientific) in 5% nonfat milk. The membranes were then incubated for 24 h with the following specific primary antibodies: anti-total STAT3 (#9139S; Cell Signaling), anti-phosphorylated STAT3 (#9145S; Cell Signaling), anti-phosphorylated AKT1/2/3 (SC-7985, Santacruz, Dallas, TX, USA), anti-total AKT1/2/3 (SC-8312, Santacruz), anti-phosphorylated ERK1/2 (#4370S; Cell Signaling), and anti-total ERK1/2 (#4695S; Cell Signaling). These membranes were left overnight at 4 °C on a rocker, diluted in 5% BSA (1:1000). We then washed the membranes with TBST and incubated the membranes with the corresponding horseradish peroxidase (HRP)-conjugated secondary antibodies for 1 h at room temperature. Using an Azure Imager c300 (Azure Biosystems, Dublin, CA, USA), we visualized the membranes. The protein band signals were quantified using the NIH ImageJ software (version 1.52r) [16].

### 2.5. Immunocytochemistry

A formaldehyde solution of 4% was used to fix immortalized leiomyoma cells following coculture and leptin treatment. Next, the cells were incubated at room temperature for 1 h with a blocking solution containing 1% PBS, 5% normal goat serum (Cell Signaling Technology, Danvers, MA, USA), and 0.3% Triton X-100 (MilliporeSigma, Burlington, MA, USA). They were then incubated for a period of overnight at 4 °C with primary antibodies against PCNA (Cell Signaling Technology; #13110), TNF-α (R&D, #MAB610-100, Minneapolis, MN, USA), TGF-β3 (Invitrogen, #PA5-32630, Waltham, MA, USA), and VEGF-A (Abcam, #ab1316, Cambridge, UK). A secondary antibody conjugated with antirabbit Alexa 488 (Thermo Fisher Scientific, #A11034) and antimouse Alexa 546 (Thermo Fisher Scientific, #A11030) was then added to the cells and incubated for 1 h in the dark at room temperature. DAPI with ProLong Gold Antifade Mountant (Thermo Fisher Scientific) were used to fix the slide overnight at room temperature. Using a Zeiss AxioPlan 2 microscope (Jena, Germany), pictures were taken at 20X magnification. Using ImageJ (version 1.52r; National Institutes of Health, Bethesda, MD, USA), we analyzed the staining intensity.

### 2.6. Statistical Analysis

We used GraphPad Prism 6.01 for statistical analysis of all the experiments. Each experiment was conducted three times. The results were expressed as the mean ± standard error of the mean (SEM). To compare two groups, we used a Student’s t-test, with differences at *p* < 0.05 considered statistically significant.

## 3. Results

### 3.1. Adipocyte Coculture and Leptin Treatment with Myometrium and Leiomyomas Cells Activates JAK2/STAT3 Signaling Pathways

**Figure 1:**
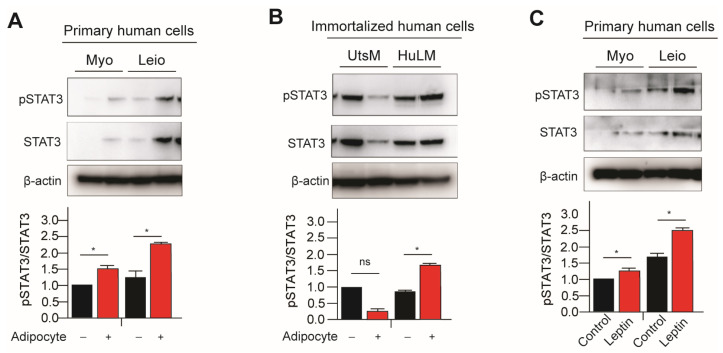
Adipocyte coculture and leptin treatment activates STAT3 signaling in myometrium and leiomyoma cells. The immortalized and primary human leiomyoma and myometrium were cocultured with adipocyte cells for eight days. Half of the medium was replaced with fresh medium every other day. After 8 days, we prepared cell lysates from the cultured cells, both with and without adipocyte coculture. Western blotting was used to analyze phosphorylated STAT3/total STAT3 protein ratios in (**A**) primary and (**B**) immortalized cells. The same Western blot membrane was probed for pSTAT3/total STAT3 and pERK1/2/total ERK1/2, presented in Figure 1 and Figure 2, respectively. Therefore, the same loading control β-actin was used for all of them. Primary human myometrium and leiomyoma cells were treated with 100 ng/mL leptin for 48 h, and Western blotting was again used to analyze phosphorylated STAT3/total STAT3 protein ratios (**C**). The mean ± SEM was determined from three independent experiments. *, *p* < 0.05 vs. the single cell type culture; ns, non-significant.

Activation of Stat3 occurs when Thy705 is phosphorylated and activated by kinases upstream, such as Janus Kinase 2 (JAK2). In our previous publication we have demonstrated that leptin treatment increased pSTAT3/STAT3 signaling in immortalized leiomyoma cells [9]. To further understand the effect of adipocyte on JAK2/STAT3 signaling in leiomyoma development, we cocultured primary and immortalized myometrium and leiomyoma cells with adipocytes and treated the leptin. Expression of phosphorylated STAT3 (pSTAT3) was significantly higher in immortalized leiomyoma cells cocultured with adipocytes and leptin-treated primary cells (Figure 1). Primary myometrium cells similarly demonstrated higher levels of pSTAT3 when cultured with adipocytes (Figure 1). No significant change in pSTAT3 was observed in immortalized myometrium cells with adipocyte coculture.

### 3.2. Leptin Treatment and Adipocyte Coculture with Myometrium and Leiomyomas Cells Activates MAPK/ERK Signaling Pathways

**Figure 2:**
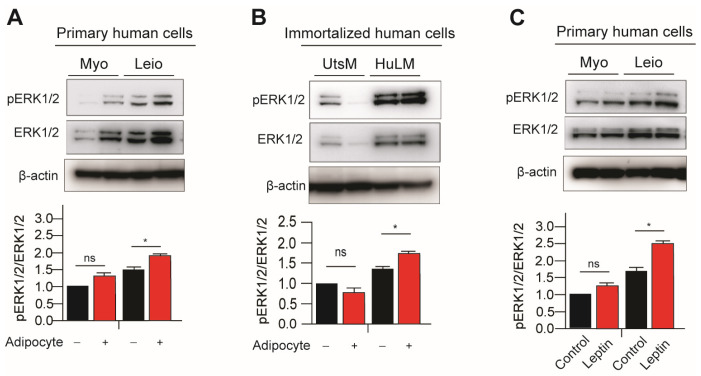
Leptin treatment and adipocyte coculture activates ERK1/2 signaling in myometrium and leiomyoma cells. For eight days, the immortalized and primary leiomyoma and myometrium cells underwent adipocyte coculture. Half of the medium was replaced with fresh medium every other day. After 8 days, we prepared cell lysates from the cultured cells, both with and without adipocyte coculture. Western blotting was used to analyze phosphorylated ERK1/2/total ERK1/2 protein ratios in (**A**) primary and (**B**) immortalized cells. The same Western blot membrane was probed for pSTAT3/total STAT3 and pERK1/2/total ERK1/2, presented in Figure 1 and Figure 2, respectively. Therefore, the same loading control β-actin was used for all of them. Primary human myometrium and leiomyoma cells were treated with 100 ng/mL leptin for 48 h, and Western blotting was used to analyze phosphorylated ERK1/2/total ERK1/2 protein ratios (**C**). The mean ± SEM was determined from three independent experiments. *, *p* < 0.05 vs. the single cell type culture; ns, non-significant.

A number of extracellular stimuli, including mitogens, growth factors, and cytokines, can activate the p44/42 MAPK (ERK1/2) signaling pathway. In order to activate p44 and p42, MEK1 and MEK2 phosphorylate and Thr202/Tyr204 and Thr185/Tyr187, respectively, are in the activation loop. The MAPK/ERK is another marker in leptin receptor signaling pathways and it was observed previously that leptin treatment increases MAPK/ERK signaling in immortalized leiomyoma cells [9]. Next, we examined the effects of adipocyte coculture or leptin treatment on MAPK/ERK signaling in immortalized and primary leiomyoma and myometrium cells. Expression of phosphorylated ERK1/2 (pERK1/2) was significantly higher in immortalized leiomyoma cells cocultured with adipocytes and leptin-treated primary leiomyoma cells (Figure 2). In primary and immortalized myometrium cells, no significant changes in phosphorylated ERK1/2 (pERK1/2) were observed.

### 3.3. Leptin Treatment and Adipocyte Coculture Further Activates PI3K/AKT Signaling in Myometrium and Leiomyoma Cells

**Figure 3:**
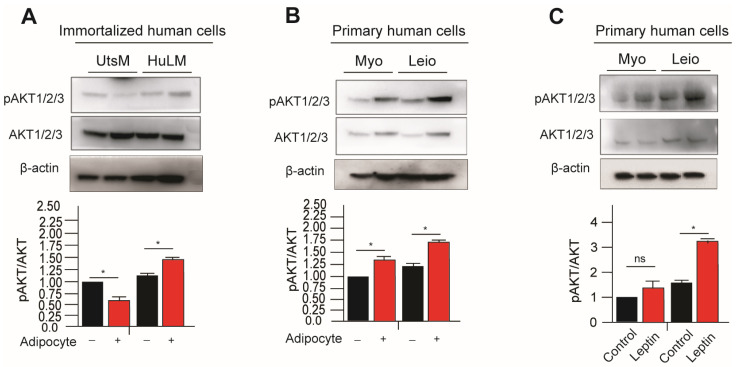
Leptin treatment and adipocyte coculture activates PI3K/AKT signaling in leiomyoma and myometrium cells. For eight days, the immortalized and primary human leiomyoma cells and myometrium and rat leiomyoma cells underwent adipocyte coculture. Every two days, 50% of the medium was exchanged with fresh medium. After eight days, cell lysates were derived from the cells cultured both with and without adipocytes. Western blotting was used to analyze phosphorylated AKT1/2/3/total AKT1/2/3 protein ratios using in (**A**) primary and (**B**) immortalized cells. Loading control was β-actin. Primary human leiomyoma and myometrium cells were treated with 100 ng/mL leptin for 48 h. Western blotting was used to analyze phosphorylated AKT1/2/3/total AKT1/2/3 protein ratios (**C**). The loading control was β-actin. The mean ± SEM was determined from three independent experiments. *, *p* < 0.05 vs. the single cell type culture; ns, non-significant.

Several members of the serine/threonine kinase AKT family exist, including AKT1, AKT2, and AKT3. The activation of AKT1 and AKT2 is dependent on the phosphatidylinositol 3-kinase complex (PI3K) subunits Thy740 and Thy751. However, both Thr 308 and Ser 473 of Akt1 are phosphorylated by insulin or insulin-growth factor-1 (IGF-1). The AKT3 protein is phosphorylated on a serine residue in response to insulin. Cells stimulated by Insulin/IGF-1 undergo phosphorylation and activation of AKT proteins. Activation of the PI3K/AKT pathway has been observed as promoting uterine leiomyoma growth [17]. In primary and immortalized leiomyoma cells, expression of phosphorylated AKT (pAKT) was significantly higher in cocultured with adipocytes and leptin-treated immortalized and primary leiomyoma cells (Figure 3). Additionally, immortalized and primary myometrium cells were found to have higher levels of pAKT when treated with leptin and cocultured with adipocytes (Figure 3).

### 3.4. Effect of Leptin on Pro-Inflammatory Cytokines in Uterine Myometrium and Leiomyoma Cells

**Figure 4:**
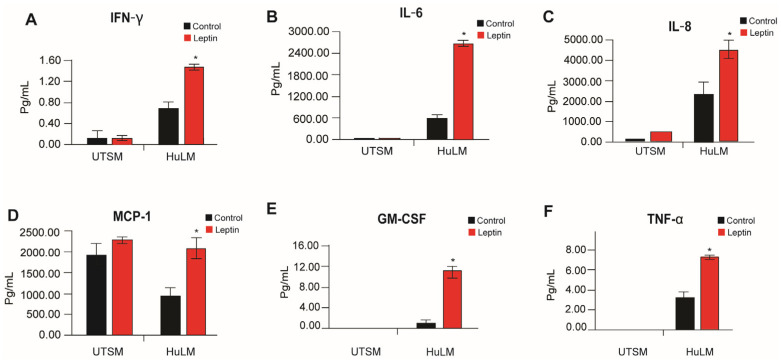
Effect of leptin treatment in inflammatory factors secretion in human immortalized leiomyoma and myometrium cells. Immortalized human leiomyoma and myometrium cells were treated with 100 ng/mL leptin for 48 h. After the incubation time, collected media were used to measure the secretion levels of (**A**) IFN-γ, (**B**) IL-6, (**C**) IL-8, (**D**) MCP-1, (**E**) GM-CSF, and (**F**) TNF-α. The data are presented as the mean ± standard error of the mean (SEM) from three independent experiments. *, *p* < 0.05 vs. control.

It is known that obesity affects the tumor microenvironment through the release of several inflammatory mediators [18]. Chronically active inflammation of the immune system appears to cause leiomyoma [19]. To investigate the effect of leptin on the pro-inflammatory cytokines, we used an enzyme-linked immunosorbent assay (ELISA) to assess the secretion of pro-inflammatory cytokines in immortalized human uterine leiomyoma (HuLM) cells and uterine smooth muscle cells (UtSM) media after treatment with leptin. Human uterine leiomyoma (HuLM) cells responded more to leptin treatment compared to myometrium cells with increased secretion of inflammatory factors: IFNγ, IL-6, IL-8, MCP-1, GM-CSF, and TNF-a (Figure 4).

### 3.5. Effect of Leptin on Angiogenic and Pro-Fibrotic Mediators in Uterine Leiomyoma and Myometrium Cells

**Figure 5:**
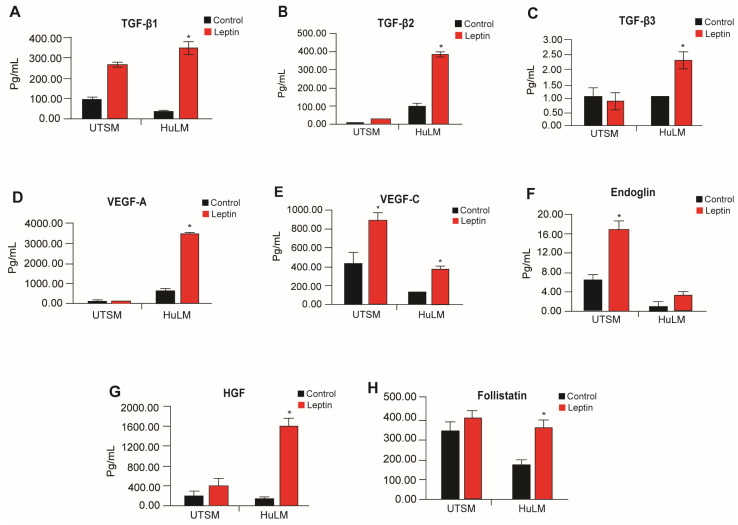
Effect of leptin treatment in fibrosis and angiogenesis factors secretion in human immortalized leiomyoma and myometrium cells. Immortalized human leiomyoma cells and myometrium were treated with 100 ng/mL leptin for 48 h. After the incubation time, the collected media were used to measure the secretion levels of (**A**) TGF-β1, (**B**) TGF-β2, (**C**) TGF-β3, (**D**) VEGF-A, (**E**) VEGF-C, (**F**) Endoglin, (**G**) HGF, and (**H**) Follistatin. The mean ± standard error of the mean (SEM) was determined from three independent experiments. *, *p* < 0.05 vs. control.

Adipocytes are known to secrete leptin, pro-fibrotic factors, and angiogenic factors [20]. Therefore, we sought to investigate whether leptin has any impact on pro-fibrotic and angiogenic mediators, specifically in the context of uterine leiomyomas. We used HuLM and UtSM cells media treated with leptin. Leptin treatment significantly increased the pro-fibrotic factors, TGF-β1, TGF-β2, and TGF-β3 and angiogenic factors, VEGF-A, HGF, and Follistatin (Figure 5). Immortalized uterine smooth muscle tissue (UtSM) cells did not show significant response to leptin treatment, with the exception of TGF-β1, VEGF-C, and Endoglin (Figure 5).

### 3.6. Effect of STAT3, ERK, and AKT Inhibitors on Leiomyoma Cells Cocultured with Adipocytes

**Figure 6:**
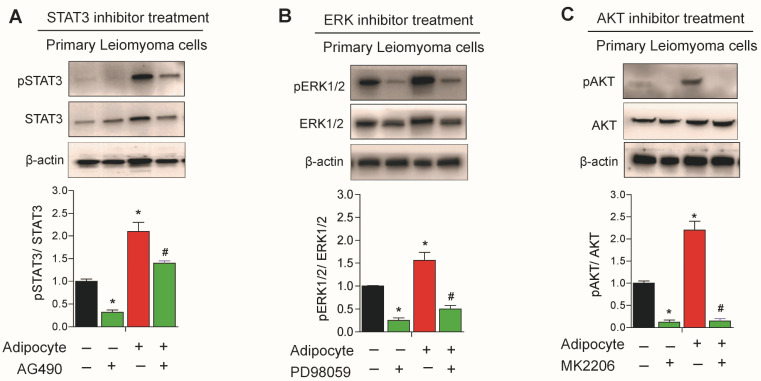
Effect of STAT3, ERK and AKT inhibitor treatment in uterine leiomyoma cells. Primary human leiomyoma cells were cultured with SW872 adipocyte cells. After seven days, primary leiomyoma cells cultured in the presence and absence of adipocytes were treated for 24 h with the STAT3 inhibitor (AG490; (**A**)), the ERK inhibitor (PD98059; (**B**)), and the AKT inhibitor (AM2206; (**C**)) before a Western blot analysis of pSTAT3/STAT3, pERK1/2/ERK1/2, and pAKT/AKT abundance. The loading control was β-actin. The mean ± SEM was determined from three independent experiments. *, *p* < 0.05 vs. the single cell type culture; #, *p* < 0.05 vs. coculture.

Leptin treatment has previously been shown to augment the effect of STAT3 and ERK inhibitors in leiomyoma cells [9]. We sought to investigate the effect of STAT3, ERK, and AKT inhibitors in leiomyoma cells cocultured with adipocytes. Leiomyoma cocultured with adipocytes significantly decreased in expression of pSTAT3, pERK1/2, and pAKT when respective inhibitors were administered (Figure 6).

### 3.7. Effect of STAT3, ERK, and AKT Inhibitors on PCNA and TNF-α Expression

**Figure 7:**
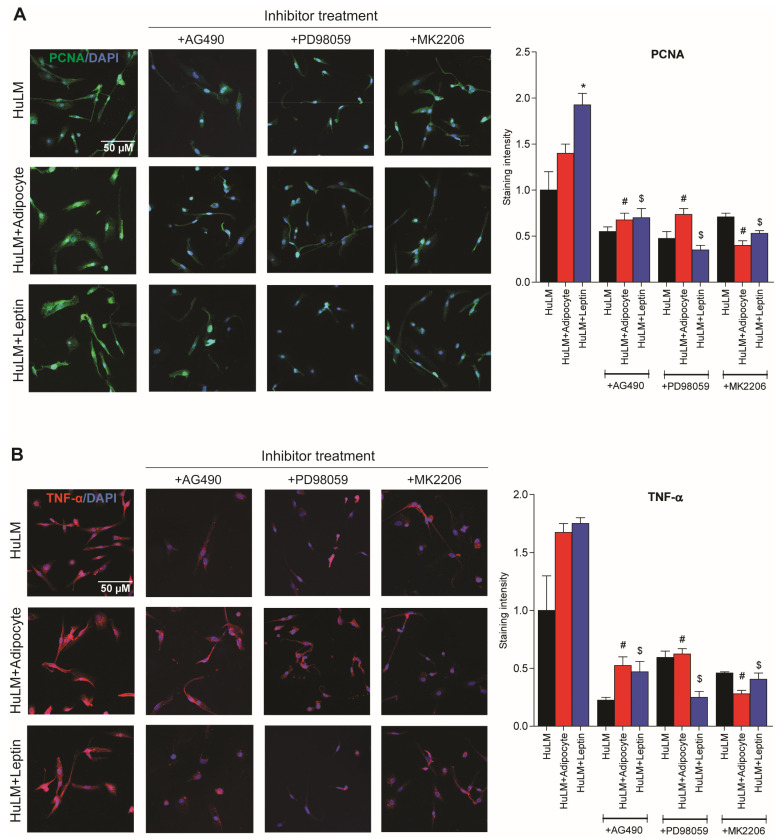
Effect of STAT3, ERK, and AKT inhibitor treatment in PCNA and TNF-α expression. Immortalized human leiomyoma cells (HuLM) were cocultured with SW872 adipocyte cells for seven days and treated with leptin for 24 h. After seven days cocultured and 24 h leptin treatment, leiomyoma cells were treated for additional 24 h with the STAT3 inhibitor (AG490), the ERK inhibitor (PD98059), and the AKT inhibitor (MK2206). We performed immunocytochemistry to confirm the presence of (**A**) PCNA (green fluorescence), (**B**) TNF-α (red fluorescence), and DAPI (blue fluorescence). All images were taken with a Zeiss AxioPlan 2 microscope system (20× magnification). Scale bar, 50 μm. Images were measured and quantified using ImageJ. *, *p* < 0.05 vs. the single cell type culture; #, *p* < 0.05 vs. coculture; $, *p* < 0.05 vs. leptin treated cells.

Adipocyte coculture and leptin treatment has previously been shown to increase PCNA expression in leiomyoma cells [9]. Elevated TNF-α expression was also observed in obese individuals [10]. We sought to investigate the effect of STAT3, ERK, and AKT inhibitors in PCNA and TNF-α expression in leiomyoma cells cocultured with adipocytes and leptin treatment. Leiomyoma cocultured with adipocytes and leptin treatment significantly increased in expression of PCNA and TNF-α (Figure 7). Furthermore, the expression was decreased when respective inhibitors were administered (Figure 7).

### 3.8. Effect of STAT3, ERK, and AKT Inhibitors on TGF-β3 and VEGF-A Expression

**Figure 8:**
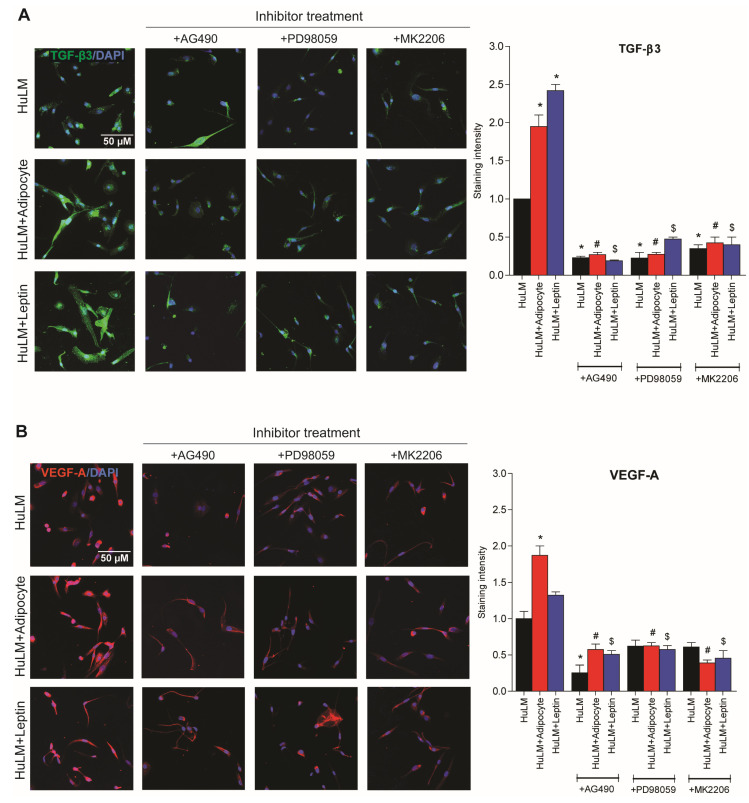
Effect of STAT3, ERK, and AKT inhibitor treatment in TGF-β3 and VEGF-A expression. Immortalized human leiomyoma cells were cocultured with SW872 adipocyte cells for seven days and treated with leptin for 24 h. After seven days cocultured and 24 h leptin treatment, leiomyoma cells were treated for additional 24 h with the STAT3 inhibitor (AG490), the ERK inhibitor (PD98059), and the AKT inhibitor (MK2206). We performed immunocytochemistry to confirm the presence of (**A**) TGF-β3 (green fluorescence), (**B**) VEGF-A (red fluorescence), and DAPI (blue fluorescence). All images were taken with a Zeiss AxioPlan 2 microscope system (20× magnification). Scale bar, 50 μm. Images were measured and quantified using ImageJ. *, *p* < 0.05 vs. the single cell type culture; #, *p* < 0.05 vs. coculture; $, *p* < 0.05 vs. leptin treated cells.

Previous studies have shown that coculture of adipocytes increases TNF-α, TGF-β3, and VEGF-A levels in medium, and TGF-β3 and VEGF-A expression in obese individuals [10]. The purpose of this study was to investigate the effect of STAT3, ERK, and AKT inhibitors on TGF-β3 and VEGF-A expression in leiomyoma cells cocultured with adipocytes and treated with leptin. As a result of coculture of leiomyoma cells with adipocytes and leptin treatment, TGF-β3 and VEGF-A expressions are significantly increased (Figure 8). Furthermore, the expression was decreased after the administration of the respective inhibitors (Figure 8).

## 4. Discussion

In this study, we focused on the impact of adipocyte coculture on leiomyoma and myometrium cells. We found increased activation of MAPK/ERK, JAK2/STAT3, and PI3k/AKT signaling pathways in human leiomyoma cells as a result of adipocyte coculture, outlining the pathophysiological link between adipocyte hypertrophy in obesity and the development of uterine leiomyoma. Additionally, inhibitors of MAPK/ERK, JAK2/STAT3, and PI3k/AKT leptin receptor pathways are more effective in leiomyoma cells cocultured with adipocytes. These findings suggest that therapeutics for uterine leiomyomas should consider the role of adipocytes in cell proliferation and growth. Finally, we found a direct impact of leptin treatment on pro-inflammatory, angiogenic, and fibrotic factors in the tumor microenvironment.

Several epidemiological studies have proven an association between obesity and uterine leiomyoma. However, the complex pathophysiology underlying this association remains unclear.

Activation of JAK2/STAT3, MAPK/ERK, and PI3k/Akt pathways have previously been linked to the binding of leptin, an adipocyte derived hormone, to Ob-R [9]; however, the effect of adipocyte coculture on uterine leiomyoma cells remained a gap in knowledge. This preliminary study elucidated the role of adipocytes in the tumor environment. For the first time, we demonstrated the amplified activation of JAK2/STAT3, MAPK/ERK, and PI3k/Akt because of adipocyte coculture along with leptin treatment in uterine leiomyoma cells. Previous studies have observed the role of obesity in promoting cancer growth, as it causes chronic inflammation and promotes cell proliferation through the secretion of leptin from adipose tissue [21]. The upregulation of the JAK2/STAT3, MAPK/ERK, and PI3k/Akt pathways impacts several pathways integral to cell growth and metabolism and implicates adipose tissue as having a role in the cancerous uterine leiomyoma growth [20]. Our study underscores the significant role of adipocytes in proliferation of uterine leiomyoma cells. Despite the increased activation of these respective pathways with adipocyte coculture, administration of JAK2/STAT3, MAPK/ERK, and PI3k/AKT inhibitors demonstrated a significant effect even with the adipocyte coculture. The respective inhibitors reduced the levels of pSTAT, pERK1/2, and pAKT and suppressed the intracellular staining intensity of PCNA, TNF-α, TGF-β3, and VEGF-A, in addition to reversing the effect of leptin treatment and adipocyte coculture on leiomyoma cell growth. Further investigation into the effect of MAPK/ERK, JAK2/STAT3, and PI3k/AKT inhibitors in adipocyte-mediated leiomyoma growth could lead to viable therapeutic strategies for uterine leiomyomas.

Our study found that leptin treatment of leiomyoma cells increased pro-inflammatory factors, MCP-1, IFNγ, IL-8, GM-CSF, IL-6, and TNF-a. Increasing evidence is demonstrating the association between leptin, an adipocyte-secreted hormone, and cancer progression [22,23,24,25,26]. Chronic inflammation of the uterine smooth muscle leads to uncontrolled ECM deposition, which promotes development of uterine leiomyomas [27]. Inflammatory factors, such as TNF-a, have been isolated at higher levels in individuals with uterine leiomyomas, highlighting the strong association between inflammation and uterine leiomyomas [28]. Understanding the mechanism through which obesity increases chronic inflammation in individuals could uncover therapeutic strategies for mitigating leiomyoma growth.

## 5. Conclusions

This study complements the body of epidemiological literature linking obesity and uterine leiomyoma growth by further elucidating the pathophysiology. Obesity has grown as an epidemic in the United States, and strategies aiming to reduce adipose tissue in individuals may help to reduce the large burden of disease of uterine leiomyomas.

## Data Availability

All data associated with this study are presented in the paper.
